# Construction of a ceRNA network in polycystic ovary syndrome (PCOS) driven by exosomal lncRNA

**DOI:** 10.3389/fgene.2022.979924

**Published:** 2022-11-04

**Authors:** Lilian Bai, Junxing Gong, Yanyan Guo, Yuchen Li, Hefeng Huang, Xinmei Liu

**Affiliations:** ^1^ International Peace Maternity and Child Health Hospital, School of Medicine, Shanghai Jiao Tong University, Shanghai, China; ^2^ Shanghai Key Laboratory of Embryo Original Disease, Shanghai, China; ^3^ Obstetrics and Gynecology Hospital, Institute of Reproduction and Development, Fudan University, Shanghai, China; ^4^ Research Units of Embryo Original Diseases, Chinese Academy of Medical Sciences, Shanghai, China

**Keywords:** ceRNA network, exosomes, granulosa cells, polycystic ovary syndrome, post-transcriptional regulation

## Abstract

Polycystic ovary syndrome (PCOS), a common and frustrating syndrome in women of reproductive age, is characterized by symptoms including hyperandrogenemia, ovulation dysfunction, and polycystic ovaries. The role of competitive endogenous RNA (ceRNA) networks is receiving increasing attention and has been reported in multiple complicated diseases, such as various carcinomas, endometriosis, and tubal factor infertility. However, the association of ceRNA networks with the pathogenesis of PCOS remains unclear. This study aimed to construct a ceRNA network orchestrated by exosomal lnRNA and circRNA in PCOS. We screened RNA data of 34 samples from the Gene Expression Omnibus (GEO) database for differentially expressed lncRNAs (DELs), miRNAs (DEMs), mRNAs (DEGs), and circRNA associated with the progression of PCOS (PCOS, *n* = 17 vs. normal, *n* = 17). A protein–protein interaction (PPI) network, gene set enrichment analysis (GSEA), and Gene Ontology (GO) and Kyoto Encyclopedia of Genes and Genomes (KEGG) analyses were conducted. Importantly, the function of the ceRNA network was explored using GO and KEGG enrichment analyses. We identified 46 DELs (25 upregulated and 21 downregulated), 31 DEMs (20 upregulated and 11 downregulated), 165 DEGs (52 upregulated and 113 downregulated), and 1 differentially expressed circRNA. The PPI network had 79 nodes and 112 edges. The GSEA results showed that these genes were mainly related to oxidative phosphorylation; TNF signaling pathways; and valine, leucine, and isoleucine degradation. GO and KEGG analyses revealed that the DEGs were significantly enriched in lipid metabolism, peroxisome proliferator-activated receptor (PPAR) signaling pathways, and fatty acid metabolism. Additionally, we constructed a novel PCOS-associated lncRNA–miRNA–mRNA ceRNA triple network and a circRNA-related network. Thereafter, we described the potential roles played by follicular fluid exosomes in PCOS. Our present study describes the molecular pathogenesis of PCOS in human ovarian granulosa cells at the post-transcriptional level, which provides new insights for the clinical diagnosis and treatment of PCOS and further scientific research.

## 1 Introduction

Polycystic ovary syndrome (PCOS) is a common disease in women and is associated with endocrine and metabolic disorders. Approximately 10.1% Chinese women of reproductive age are affected by PCOS (Wu et al., 2021). The disease has two typical characteristics: androgen excess (hyperandrogenism) and ovarian dysfunction [oligoovulation and/or polycystic ovary pattern (PCOM)]. In addition, most patients exhibit significant metabolic abnormalities, mainly insulin resistance and compensatory hyperinsulinemia (Ezeh et al., 2021). The most widely used classification of PCOS is the Rotterdam definition, which is currently supported by most scientific and health sectors (Legro et al., 2013). The definition suggests that any woman with at least two of the following three diseases can be diagnosed with PCOS: clinical or biochemical hyperandrogenism, ovulation disorders, and PCOM (Rotterdam ESHRE/ASRM-Sponsored PCOS Consensus Workshop Group, 2004). PCOS is caused by various factors, including androgen imbalance, increased luteinizing hormone (LH) levels, inflammatory response, and oxidative stress, and its pathophysiological mechanism is complex (Li et al., 2017).

Granulosa cells, a layer of cells occurring on the surface of follicles, are an important component of the ovary. Mounting evidence indicates that the disruption of follicle development and ovulation in PCOS is associated with granulosa cell dysfunction (Liu et al., 2022; Zhang et al., 2022). In addition, mural granulosa cells participate in oocyte meiosis arrest by secreting oocyte maturation inhibitory factor (OMI) (Kawamura et al., 2011). Studies have also found that granulosa cell proliferation is increased in PCOS patients (Bao et al., 2021). Additionally, the abnormal proliferation of granulosa cells may increase the number of preantral follicles and ovarian volume, which may lead to the formation of large follicles in the ovaries of PCOS patients. Therefore, the abnormal state of granulosa cells is associated with the occurrence of PCOS, suggesting that improving the abnormal state of granulosa cells has potential therapeutic value.

Exosomes are membrane-wrapped vesicles, 30–150 nm in diameter, and are secreted by multiple cells into the extracellular microenvironment. They contain various types of nucleic acids, including mRNA, microRNA (miRNA), and long non-coding RNA (lncRNA), and are indispensable for extracellular communication (Kalluri and LeBleu, 2020; Donoso-Quezada et al., 2021). Recent literature shows that exosomes can be synthesized and released in different parts of the female reproductive tract, such as in the tubal epithelial cells, endometrium, uterus, and follicular fluid, which is the key microenvironment for follicular development (Machtinger et al., 2016; Gurung et al., 2020; Li et al., 2020a; Giacomini et al., 2021). Exosomes can transmit information to granulosa cells (Pegtel and Gould, 2019). Abnormalities in either exosomes or granulosa cells play an important role in the development of PCOS. However, the mechanism of communication between exosomes and granulosa cells, as well as their therapeutic targets, have not been well understood. Therefore, an in-depth exploration of exosomes and granulosa cells and their role in the occurrence and development of PCOS is required.

The vast majority of mammalian genomes are transcribed as non-coding RNA, for instance, miRNA, lncRNA, and circular RNA (circRNA) (Yao et al., 2019). Recently, lncRNA has captured the attention of many scientists. A study on the lncRNA of follicular fluid exosomes using high-throughput sequencing screened 1,866 differentially expressed lncRNAs. Functional analysis showed that they were closely related to pathways such as endocytosis, Hippo, and MAPK. This suggests that lncRNAs are important in the pathogenesis of PCOS (Wang et al., 2020). Studies have identified differential expression profiles of lncRNAs and mRNAs in exosomes and have revealed some biological processes and regulatory networks related to PCOS (Zhou et al., 2022). CircRNAs are covalently closed structures (Chen, 2016). At present, a few studies have found that circRNAs are involved in the occurrence and development of PCOS. For example, Jia et al. reported that circ_0118530 is increased in PCOS patients (Jia et al., 2020).

miRNAs consist of 9–25 nucleotides (Wang et al., 2015) and are also crucial for the development of PCOS (Abdalla et al., 2020; Bao et al., 2021). For example, one study found that miR-200b expression was significantly increased in PCOS patients compared to that in controls. Moreover, the proliferation of a granulosa-like tumor cell line was inhibited by the overexpression of miR-200b and miR-200c (He et al., 2019). The expression of miR-93 and miR-21 was increased in the granulosa cells of hyperandrogenic PCOS patients, and the expression of miR-93 and miR-21 was positively correlated with free testosterone and free androgen index (Tu et al., 2019). However, different sequencing platforms, different statistical methods, and small sample sizes can all lead to erratic study results. Further comprehensive analyses are necessary to reliably identify the molecular mechanisms of disease development.

The theory about competitive endogenous RNA (ceRNA) was first put forward by Salmena et al. (2011). Subsequently, experiments have confirmed that some RNAs act as ceRNA, such as *cIRS-7* (also known as *CDR1-AS*, a circRNA) regulating the activity of miR-7 in the central nervous system (Memczak et al., 2013). The ability of pseudogenes, such as *PTENP1,* to act as ceRNAs has also been experimentally demonstrated (Yu et al., 2014). Furthermore, lncRNAs can interact with miRNAs, messenger RNAs (mRNAs), and proteins for performing their biological functions. Additionally, lncRNAs act as ceRNAs that communicate with mRNAs through competitively shared miRNAs (Ergun and Oztuzcu, 2015; Piao et al., 2020); the specific mechanism is as follows. When ceRNA is transcriptionally silenced, the parental mRNA is transcribed and exported to the cytoplasm, where it is targeted by the microRNA-guided RNA-induced silencing complex (miRNA-RISC), resulting in accelerated degradation, translation blockade, and reduced expression. When the ceRNA with competing target sites becomes transcriptional active, it will compete for miRNA targeting and binding to the RISC complex. This isolates the miRNA-RISC complex from the parent gene and results in the increased expression of the parent gene (Thomson and Dinger, 2016).

**FIGURE 1:**
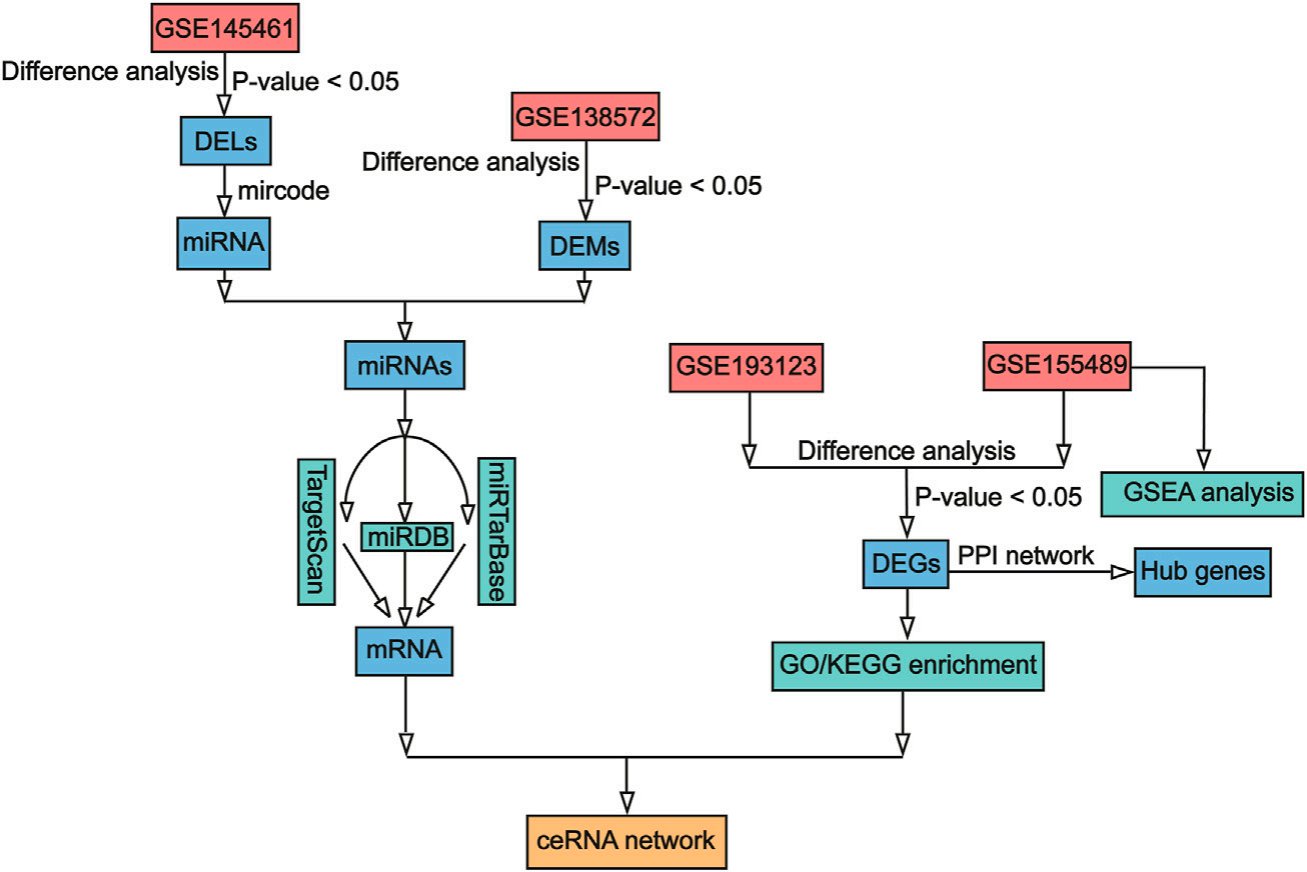
Workflow of data processing and analysis. DEL, Differentially expressed lncRNA; DEM, differentially expressed miRNA; DEG, differentially expressed mRNA; lncRNA, long non-coding RNA; miRNA, microRNA; mRNA, messenger RNA; GO, Gene Ontology; KEGG, Kyoto Encyclopedia of Genes and Genomes; GSEA, gene set enrichment analysis; ceRNA, competing endogenous RNA.

The role of ceRNA networks is receiving increasing attention and has been reported in several complicated diseases, including carcinomas (Chen et al., 2022; Xiong et al., 2022), endometriosis (Li et al., 2022), and tubal factor infertility (Li J. Z. et al., 2021a). Some previous studies have constructed ceRNA networks for the development of PCOS. For example, a study constructed an lncRNA–mRNA network associated with PCOS on the basis of granulosa cells, and key lncRNAs were found to be significantly associated with processes and pathways related to inflammation, oxidative stress, and apoptosis (Ma et al., 2021). Other studies have also constructed ceRNA networks and identified ceRNA axes such as lncRNAs miR-483-5p-GOT2; most of the axes are closely associated with steroid biosynthesis and metabolic pathways (Zhao et al., 2021). However, few studies have investigated PCOS-associated lncRNA–miRNA–mRNA ceRNA triple networks between follicular fluid and granulosa cells. In the current study, to explore the possible mechanisms of PCOS pathogenesis in human ovarian granulosa cells at the post-transcriptional level, we conducted investigations (described in the flowchart in Figure 1 and Supplementary Figure S1), identified DELs in exosomes and DEMs and DEGs in granulosa cells, and constructed an integrated analysis of the lncRNA–miRNA–mRNA ceRNA network.

## 2 Materials and methods

### 2.1 Data collection

We searched the Gene Expression Omnibus (GEO) database for datasets related to PCOS and exosomes using the keywords “polycystic ovary syndrome” and “exosomes” and downloaded four datasets, namely, GSE145461, GSE138572, GSE193123, and GSE155489. To obtain data on lncRNA and circRNA expressions in the follicular fluid exosomes of patients with PCOS, the GSE145461 dataset (platform: GPL20301 Illumina HiSeq 4,000) was used. This dataset had been obtained by deep sequencing of the transcriptome of follicular fluid from five PCOS patients and five control patients using Illumina GAIIx. To obtain data on miRNA expression in granulosa cells of patients with PCOS, the GSE138572 dataset was used (platform: GPL11154 Illumina HiSeq, 2000). This dataset had been developed using ovarian granulosa cells from five PCOS patients and five healthy volunteers. The dataset GSE193123 (platform: GPL24676 Illumina NovaSeq 6,000) contains gene/mRNA expression data obtained from ovarian granulosa cells from three healthy women and three women with PCOS. The dataset GSE155489 (platform: GPL20795 HiSeq X Ten) contains gene/mRNA expression data obtained from granulosa cells from four healthy women and four women with PCOS.

### 2.2 Identification of differentially expressed genes

Differentially expressed lncRNAs (DELs) in exosome samples and differentially expressed miRNAs (DEMs) and differentially expressed mRNAs (DEGs) in granulosa cells between patients with PCOS and healthy individuals were screened using DEseq2 R packages (Love et al., 2014). Differentially expressed circRNAs in exosome samples between patients with PCOS and healthy individuals were screened using limma and edgeR packages (Ritchie et al., 2015), respectively. For all screening analyses, *p*-value < 0.05 was deemed statistically significant.

### 2.3 Construction of a protein–protein interaction network

We used STRING 11.0 (https://string-db.org/), an online database, to study important genes associated with PCOS in granulosa cells. A PPI network on DEGs was constructed with a confidence score ≥ 0.4. We generated the results using Cytoscape software (V2.8.3) (Shannon et al., 2003).

### 2.4 Functional enrichment analysis

To explore the possible biological role of granulosa cells in PCOS, GSEA was performed by the clusterProfiler R package. Moreover, to forecast the possible functions of these DEGs, Kyoto Encyclopedia of Genes and Genomes (KEGG) pathway (Kanehisa et al., 2012) and Gene Ontology (GO) analyses of DEGs were conducted using the clusterProfiler R package. GO analysis was conducted from three distinct aspects: biological process (BP), cellular component (CC), and molecular function (MF). *p*-value < 0.05 was considered statistically significant. To predict the function of these DEGs with high accuracy, we also performed GO analysis and KEGG analysis on DEGs using ClueGO v 2.5.8. ClueGO is a plugin for Cytoscape that allows for the classification of non-redundant GO terms into categories and the visualization of functionally related genes in a clustering network (Bindea et al., 2009). The threshold was set at *p*-value < 0.05.

### 2.5 Construction of lncRNA–miRNA–mRNA ceRNA network

lncRNA–miRNA pairs and miRNA–mRNA pairs can form lncRNA–miRNA–mRNA triplets. miRNA can bind to targeted mRNA to promote the degradation of mRNA, whereas lncRNA can bind to targeted miRNA to inhibit the degradation of mRNA. Here, we used the ggalluvial R package (Rosvall and Bergstrom, 2010) to construct lncRNA–miRNA–mRNA triplets on the basis of DEGs and DELs through miRcode v 11(Jeggari et al., 2012; http://www.mircode.org/mircode/), miRDB v 7.0 (Wong et al., 2015; http://mirdb.org/), miRTarBase (Huang et al., 2020; https://mirtarbase.cuhk.edu.cn/∼miRTarBase/miRTarBase_2022/php/index.php), and TargetScan v 7.2 (Enright et al., 2003; http://targetscan.org/vert_72/).

First, we predicted the lncRNA–miRNA pairs through the miRcode database on the base of DELs. miRcode is a human microRNA target prediction database based on GENCODE gene annotation. Then, we used three databases, miRDB, miRTarBase, and TargetScan, to predict the target genes of miRNAs. miRDB is an online database for target prediction and functional annotation of miRNAs through the MirTarget bioinformatics tool. miRTarBase is a microRNA target database with experimentally confirmed data, and TargetScan is a software for predicting miRNA binding sites in mammals. We set the parameters such that genes appearing in any two of these databases were considered target genes for these miRNAs. Subsequently, we compared the predicted target genes with DEGs and used overlapping genes and the pairs of roles corresponding to them to build lncRNA–miRNA–mRNA triplets (ceRNA network).

### 2.6 GO and KEGG enrichment analysis of the ceRNA network

To further understand the function of genes in the ceRNA network, we accomplished GO and KEGG path analyses with the clusterProfiler R package on our ceRNA network to predict the function of this network. *p*-value < 0.05 was considered statistically significant.

### 2.7 CircRNA-related network

First, considering the differentially expressed circRNAs, we predicted the circRNA–miRNA pairs using the circinteractome (Dudekula et al., 2016) and the cancer-specific circRNA database (CSCD) (Xia et al., 2018). We then set the parameters such that genes appearing in two of these databases were considered target miRNAs for these circRNAs. Subsequently, we used TargetScan, miRDB, and miRTarBase databases to predict mRNAs as previously described to build the circRNA–miRNA–mRNA network.

## 3 Results

### 3.1 Identification of differentially expressed genes

**FIGURE 2:**
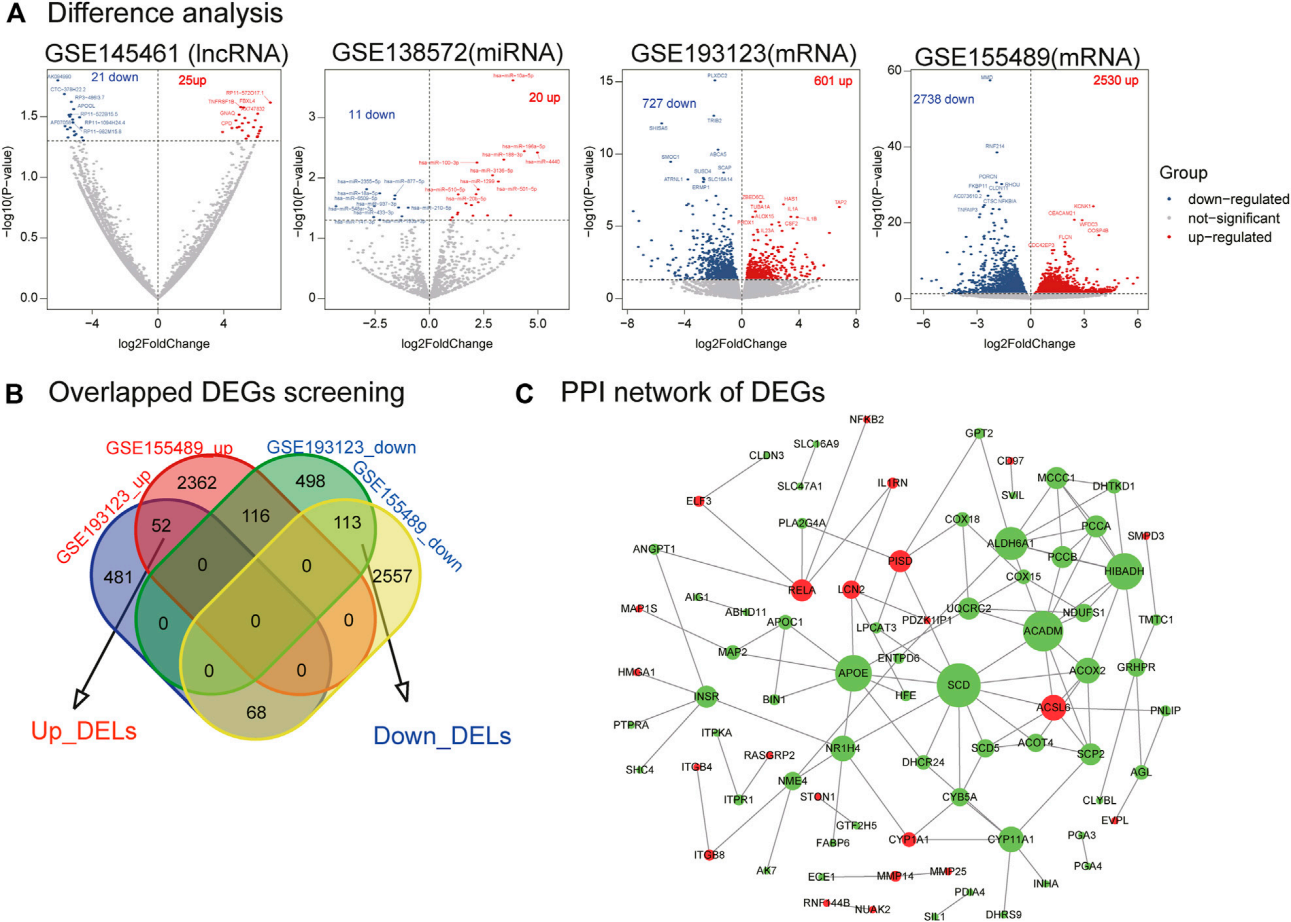
Differential analysis of lncRNA, miRNA, mRNA, mRNA crossover genes, and PPI network analysis. **(A)** Difference analysis. Volplot of DELs, DEMs, and DEGs. **(B)** Overlapped DEGs screening. **(C)** PPI network of DEGs. Red nodes represent upregulated genes in PCOS, and green nodes represent downregulated genes in PCOS.

We identified 46 DELs (25 upregulated and 21 downregulated) in GSE145461 (Figure 2A). Additionally, 31 DEMs (20 upregulated and 11 downregulated) were identified in GSE138572. Further, 1,328 differentially expressed mRNAs (601 upregulated and 727 downregulated) were identified in GSE193123, and 5,268 differentially expressed mRNAs (2,530 upregulated and 2,738 downregulated) were identified in GSE155489. Next, 165 DEGs (52 upregulated and 113 downregulated) were identified (Figure 2B). However, only one differentially expressed circRNA was identified (Supplementary Tables S1, S2, Supplementary Figure S2). The identified DELs, DEMs, and DEGs were used in the next analysis.

### 3.2 PPI network construction

We utilized the STRING database to build the PPI network of DEGs. One hundred and sixty-five DEGs were delivered to the STRING database, and 79 genes were mapped to the PPI network. The PPI network of DEGs contained 79 nodes and 112 edges, and their PPI enrichment *p*-values were below 0.05 (Figure 2C). In this network, several important genes are downregulated (e.g., *SCD*, *ACADM*, and *APOE*), whereas only a few genes are upregulated (e.g., *ACSL6*, *RELA*, and *PISD*).

### 3.3 Functional enrichment analysis

**FIGURE 3:**
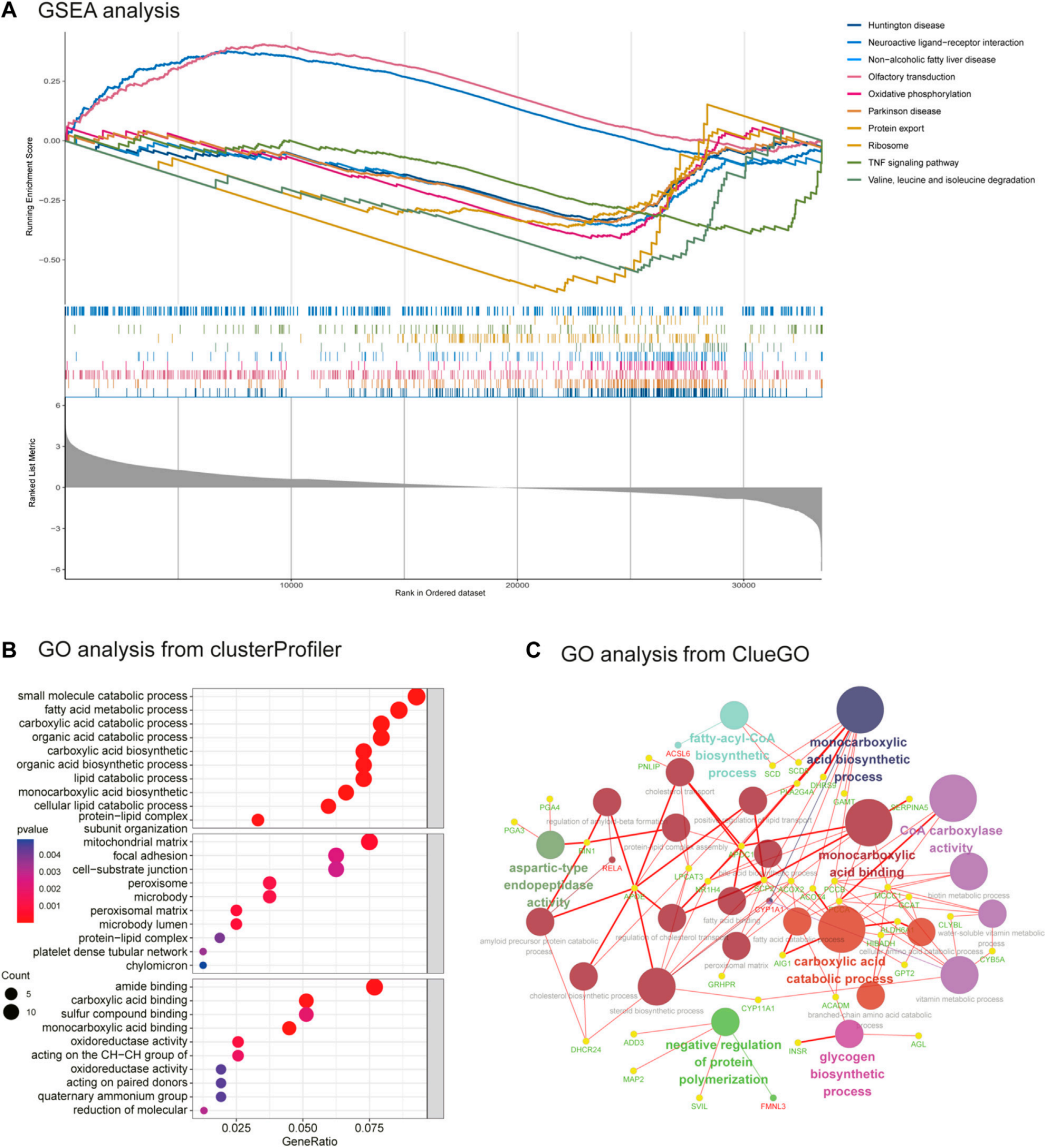
GSEA enrichment analysis and GO analysis from ClueGO. **(A)** GSEA analysis. **(B)** GO analysis of DEGs from clusterprofiler. **(C)** GO analysis of DEGs from ClueGO (version: v.2.5.8).

**FIGURE 4:**
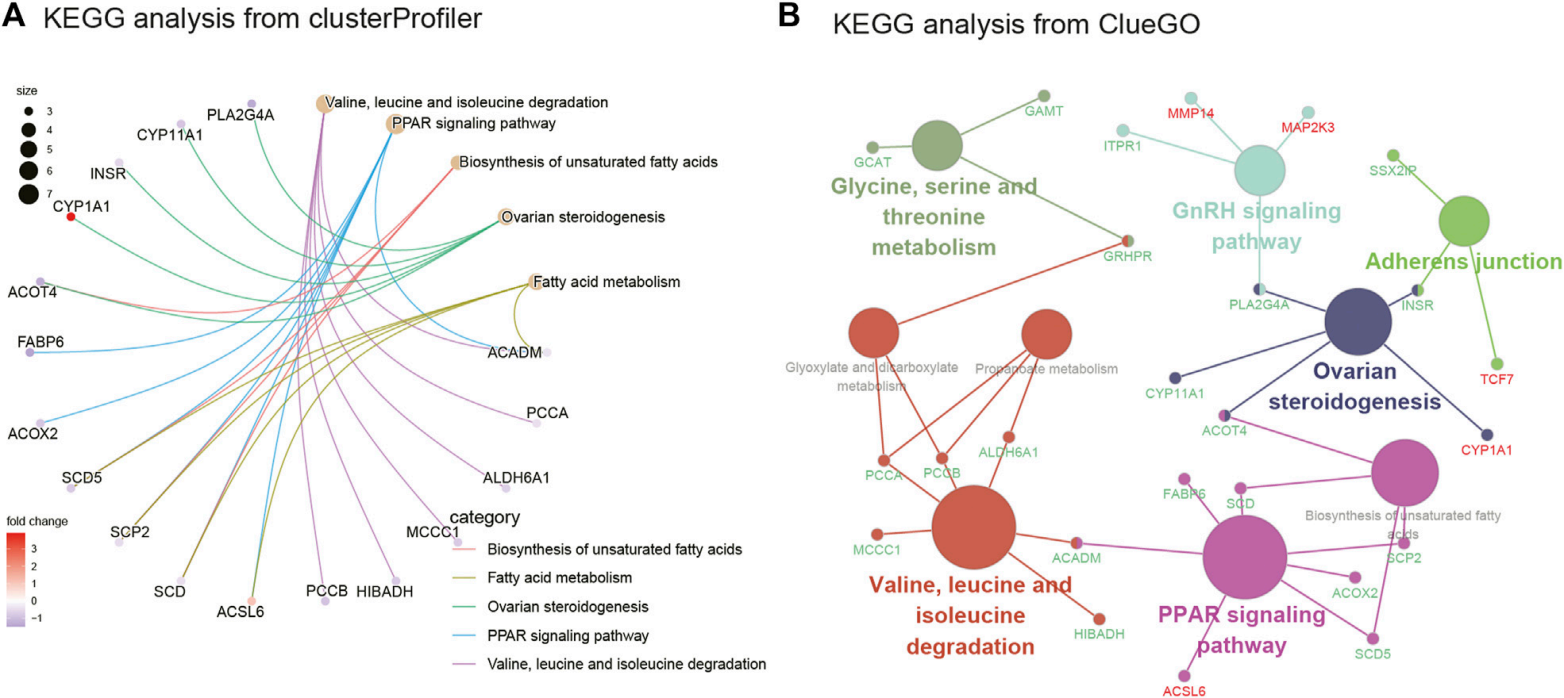
KEGG enrichment analysis and KEGG analysis from ClueGO. **(A)** KEGG analysis of DEGs from clusterprofiler. **(B)** KEGG analysis of DEGs from ClueGO (version: v.2.5.8).

As seen in Figure 3A, GSEA showed that these genes were mainly associated with the oxidative phosphorylation pathway; TNF signaling pathway; and valine, leucine, and isoleucine degradation. As illustrated in Figure 3B, Figure 3C, and Supplementary Tables S1, the results of the GO analysis demonstrated that the BP of DEGs was significantly enriched in carboxylic acid decomposition, organic acid catabolism, small-molecule catabolism, monocarboxylic acid biosynthesis, and fatty acid metabolism. Changes in the CC of DEGs were mainly observed in the mitochondrial matrix, peroxisomal matrix, microbody lumen, peroxisome, and microbody. Changes in the MF were mainly observed in monocarboxylic acid, carboxylic acid, amide, and peptide hormone binding and in oxidoreductase activity, acting on the CH-CH group of donors. GO analysis from ClueGO showed that DEGs were mainly enriched in the monocarboxylic acid biosynthetic process, CoA carboxylase activity, monocarboxylic acid binding, and carboxylic acid catabolic process. Similarly, KEGG pathway analysis revealed that the DEGs were primarily enriched in valine, leucine, and isoleucine degradation; the PPAR signaling pathway; biosynthesis of unsaturated fatty acids; ovarian steroidogenesis; and fatty acid metabolism. KEGG analysis from ClueGO indicated that the DEGs were mainly enriched in valine, leucine, and isoleucine degradation; the PPAR signaling pathway; ovarian steroidogenesis; adherens junction; GnRH signaling pathway; and glycine, serine, and threonine metabolism (Figure 4 and Table 1).

**TABLE 1:** KEGG analysis of DEGs.

ID	Description	*p* Value	Gene ID	Count
hsa00280	Valine, leucine, and isoleucine degradation	9.54E-06	ACADM/PCCA/ALDH6A1/MCCC1/HIBADH/PCCB	6
hsa03320	PPAR signaling pathway	1.20E-05	ACSL6/ACADM/SCD/SCP2/SCD5/ACOX2/FABP6	7
hsa01040	Biosynthesis of unsaturated fatty acids	0.0001638	SCD/SCP2/SCD5/ACOT4	4
hsa04913	Ovarian steroidogenesis	0.0001803	CYP1A1/INSR/CYP11A1/ACOT4/PLA2G4A	5
hsa01212	Fatty acid metabolism	0.0003059	ACSL6/ACADM/SCD/SCP2/SCD5	5
hsa04936	Alcoholic liver disease	0.0036389	TCF7/MAP2K3/RELA/ACADM/SCD/SCD5	6
hsa00630	Glyoxylate and dicarboxylate metabolism	0.0036728	PCCA/PCCB/GRHPR	3
hsa00640	Propanoate metabolism	0.00442	PCCA/ALDH6A1/PCCB	3
hsa00260	Glycine, serine, and threonine metabolism	0.0082913	GRHPR/GCAT/GAMT	3
hsa04014	Ras signaling pathway	0.0106356	RASGRP2/RELA/INSR/PAK5/ANGPT1/PLA2G4A/SHC4	7
hsa00120	Primary bile acid biosynthesis	0.0133191	SCP2/ACOX2	2
hsa04912	GnRH signaling pathway	0.0161708	MMP14/MAP2K3/ITPR1/PLA2G4A	4
hsa01200	Carbon metabolism	0.0322481	PCCA/ALDH6A1/PCCB/GPT2	4
hsa04010	MAPK signaling pathway	0.0339868	RASGRP2/NFKB2/MAP2K3/RELA/INSR/ANGPT1/PLA2G4A	7
hsa04714	Thermogenesis	0.0344138	ACSL6/MAP2K3/UQCRC2/NDUFS1/COX15/COX18	6
hsa04520	Adherens junction	0.0380892	TCF7/INSR/SSX2IP	3
hsa05412	Arrhythmogenic right ventricular cardiomyopathy	0.0466402	ITGB4/ITGB8/TCF7	3

### 3.4 ceRNA network of lncRNA–miRNA–mRNA construction

**FIGURE 5:**
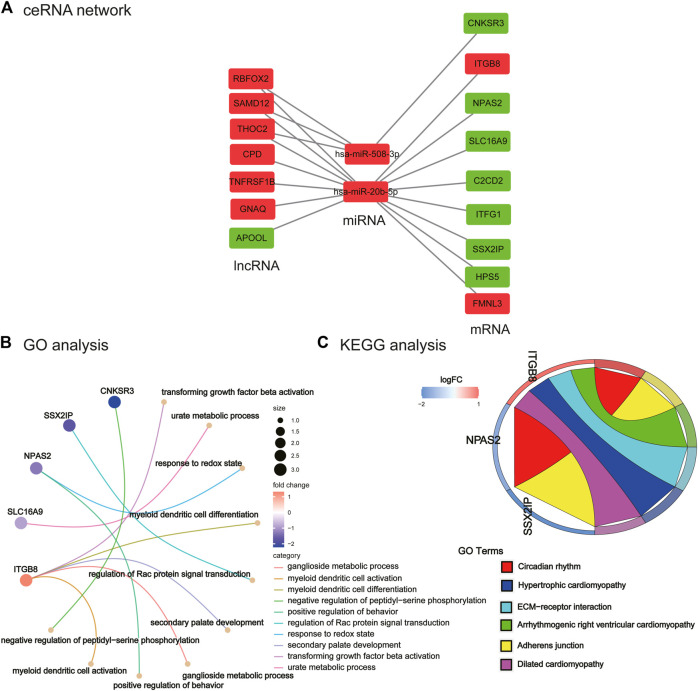
Construction of ceRNA network and its functional enrichment analysis. **(A)** ceRNA network. Red nodes represent upregulated lncRNA/miRNA/mRNA in PCOS, and green nodes represent downregulated lncRNA/miRNA/mRNA genes in PCOS. **(B)** GO analysis of ceRNA network. **(C)** KEGG analysis of ceRNA network.

As illustrated in Figure 5A, a ceRNA network was constructed including seven lncRNAs (e.g., SAMD12 and GNAQ), two miRNAs (hsa-miR-508-3p and hsa-miR-20b-5p), and nine mRNAs (e.g., CNKSR3, ITGB8, and HPS5). In the ceRNA network that we mapped, we found seven lncRNAs, of which six were upregulated and one was downregulated. The two identified miRNAs identified were upregulated. Of the nine mRNAs identified, two were upregulated and seven were downregulated. These results suggest that the lncRNA carried by exosomes may regulate the expression of some mRNAs in PCOS by hsa-miR-508-3p and hsa-miR-20b-5p.

### 3.5 Functional enrichment analysis of ceRNA network

We performed enrichment analysis of GO function and the KEGG pathway. The top 10 enriched GO items are listed in Figure 5B and Table 2. GO analysis revealed that the ceRNA network was significantly enriched in transforming growth factor beta activation, urate metabolic process, response to the redox state, myeloid dendritic cell differentiation, regulation of Rac protein signal transduction, secondary palate development, ganglioside metabolic process, positive regulation of behavior, myeloid dendritic cell activation, and negative regulation of peptidyl-serine phosphorylation. KEGG pathway enrichment analysis was further conducted for this ceRNA network. As listed in Figure 5C and Table 3 (all six signal pathways), this ceRNA network suggested significant enrichment in the circadian rhythm, adherens junction, ECM–receptor interaction, etc.

**TABLE 2:** GO analysis of ceRNA network.

Ontology	ID	Description	*p* value	Gene ID	Count
BP	GO:0036363	Transforming growth factor beta activation	0.003733329	ITGB8	1
BP	GO:0046415	Urate metabolic process	0.004850996	SLC16A9	1
BP	GO:0051775	Response to redox state	0.005595509	NPAS2	1
BP	GO:0043011	Myeloid dendritic cell differentiation	0.007454705	ITGB8	1
BP	GO:0035020	Regulation of Rac protein signal transduction	0.007826187	SSX2IP	1
BP	GO:0062009	Secondary palate development	0.009310921	ITGB8	1
BP	GO:0001573	Ganglioside metabolic process	0.009681807	ITGB8	1
BP	GO:0048520	Positive regulation of behavior	0.010052574	NPAS2	1
BP	GO:0001773	Myeloid dendritic cell activation	0.01079375	ITGB8	1
BP	GO:0033137	Negative regulation of peptidyl-serine phosphorylation	0.01116416	CNKSR3	1
BP	GO:0060674	Placenta blood vessel development	0.01116416	ITGB8	1
BP	GO:0001662	Behavioral fear response	0.012644611	NPAS2	1
BP	GO:0002209	Behavioral defense response	0.013014427	NPAS2	1
BP	GO:0010765	Positive regulation of sodium ion transport	0.013014427	CNKSR3	1
BP	GO:0042596	Fear response	0.014123162	NPAS2	1
BP	GO:0030866	Cortical actin cytoskeleton organization	0.014861726	FMNL3	1
BP	GO:0042073	Intraciliary transport	0.014861726	SSX2IP	1
BP	GO:0071634	Regulation of transforming growth factor beta production	0.01523083	ITGB8	1
BP	GO:0016601	Rac protein signal transduction	0.015599815	SSX2IP	1
BP	GO:0071604	Transforming growth factor beta production	0.015968682	ITGB8	1
BP	GO:0097028	Dendritic cell differentiation	0.017442965	ITGB8	1
BP	GO:0006687	Glycosphingolipid metabolic process	0.020753181	ITGB8	1
BP	GO:0030865	Cortical cytoskeleton organization	0.022588056	FMNL3	1
BP	GO:0032922	Circadian regulation of gene expression	0.025151929	NPAS2	1
BP	GO:0033555	Multicellular organismal response to stress	0.026248966	NPAS2	1
BP	GO:0050795	Regulation of behavior	0.026248966	NPAS2	1
BP	GO:0033627	Cell adhesion mediated by integrin	0.02661441	ITGB8	1
BP	GO:0045739	Positive regulation of DNA repair	0.026979736	NPAS2	1
BP	GO:0070373	Negative regulation of ERK1 and ERK2 cascade	0.028804605	CNKSR3	1
BP	GO:0001570	Vasculogenesis	0.029533731	ITGB8	1
BP	GO:0060021	Roof of mouth development	0.031354492	ITGB8	1
BP	GO:0002028	Regulation of sodium ion transport	0.033172325	CNKSR3	1
BP	GO:0043473	Pigmentation	0.036074776	HPS5	1
BP	GO:0006664	Glycolipid metabolic process	0.03679922	ITGB8	1
BP	GO:1903509	Liposaccharide metabolic process	0.037161267	ITGB8	1
BP	GO:0006672	Ceramide metabolic process	0.037523198	ITGB8	1
BP	GO:2001022	Positive regulation of response to DNA damage stimulus	0.03860829	NPAS2	1
BP	GO:0007229	Integrin-mediated signaling pathway	0.039331102	ITGB8	1
BP	GO:0015718	Monocarboxylic acid transport	0.040775329	SLC16A9	1
BP	GO:0006282	Regulation of DNA repair	0.047610021	NPAS2	1
BP	GO:0007098	Centrosome cycle	0.047610021	SSX2IP	1
CC	GO:0031082	BLOC complex	0.006427894	HPS5	1
CC	GO:0008305	Integrin complex	0.0141838	ITGB8	1
CC	GO:0098636	Protein complex involved in cell adhesion	0.016454684	ITGB8	1
CC	GO:0034451	Centriolar satellite	0.048203228	SSX2IP	1
MF	GO:0032794	GTPase activating protein binding	0.005703407	FMNL3	1
MF	GO:0051879	Hsp90 protein binding	0.016650969	NPAS2	1
MF	GO:0008028	Monocarboxylic acid transmembrane transporter activity	0.021898912	SLC16A9	1
MF	GO:0031072	Heat shock protein binding	0.048145526	NPAS2	1

**TABLE 3:** KEGG analysis of ceRNA network.

ID	Description	*p* value	Gene ID	Count
hsa04710	Circadian rhythm	0.011419339	NPAS2	1
hsa04520	Adherens junction	0.02602507	SSX2IP	1
hsa05412	Arrhythmogenic right ventricular cardiomyopathy	0.028203443	ITGB8	1
hsa04512	ECM–receptor interaction	0.032188685	ITGB8	1
hsa05410	Hypertrophic cardiomyopathy	0.032912102	ITGB8	1
hsa05414	Dilated cardiomyopathy	0.03508019	ITGB8	1

### 3.6 Construction of circRNA-related network

We used circinteractome and CSCD database to predict miRNAs of differentially expressed circRNA, and only two miRNAs were found after intersection (Supplementary Tables S3, S4, Supplementary Figure S3). However, both these miRNAs had no intersection with DEM (Supplementary Figure S4). We then used miRDB, miRTarBase, and TargetScan database to predict the mRNA of the two miRNAs identified (Supplementary Table S5). The target mRNA and DEG had an intersection gene, HMGA1 (Supplementary Figure S5). Using the results obtained, we constructed the circRNA-related network (Supplementary Figure S6).

## 4 Discussion

Exosomes are a newly discovered component of the follicular fluid in ovaries. They deliver miRNAs, mRNAs, and proteins related to ovarian follicle development and oocyte maturation (Uzbekova et al., 2020; Cao et al., 2022). Exosomes in the follicular fluid can transmit genetic information to target cells, thereby changing the function of target cells, which is a novel mechanism for explaining the etiology of PCOS (Pegtel and Gould, 2019). As one of the “goods” delivered by exosomes, lncRNAs potentially play various important regulatory roles in the pathogenesis of PCOS (Tu et al., 2021). Importantly, in the ceRNA hypothesis, lncRNAs are called miRNA sponges because they can bind miRNAs and prevent miRNA function, and lncRNAs take advantage of this by playing a regulatory role (Tan et al., 2015; Duan et al., 2020). Therefore, we need to further explore the relationship between the lncRNAs of exosomes and the miRNA and mRNA in granulosa cells to understand the disease progression of PCOS. Some studies have targeted exosomes or granulosa cells to construct ceRNA networks. The novelty of our study is that it is based on the construction of ceRNA networks employing lncRNA in exosomes and miRNA and mRNA in granulosa cells. We found that the pathway was mainly enriched in the TNF signaling pathway, PPAR signaling pathway, fatty acid metabolism, circadian rhythm, etc.

The PPI networks proposed that some key genes, such as *SCD*, *APOE*, and *ACADM*, were downregulated in patients with PCOS. From the KEGG analysis graph plotted by ClueGO, we can see that SCD is in the PPAR pathway, and ACADM is in the PPAR pathway and valine, leucine, and isoleucine degradation pathway. SCD in humans has two subtypes and is closely associated with lipid metabolism and insulin resistance. One study used non-targeted metabolomics to study the serum metabolic profile and found increased stearoyl coenzyme A desaturase (SCD) activity in PCOS patients (Zhao et al., 2014). However, we analyzed genes from granulosa cells showing reduced SCD expression. Gene expression in different tissues cannot be compared directly. The APOE ε2 allele appears to be associated with abdominal obesity, insulin resistance, and metabolic syndrome in a group of women from southwest China who have PCOS (Liu et al., 2013). The *ACADM* gene encodes a medium-chain acyl coenzyme A dehydrogenase, which is involved in mitochondrial medium-chain fatty acid β-oxidation. At present, little research has focused on *SCD*, *APOE*, and *ACADM* in PCOS. Therefore, these genes are relatively recently discovered molecules in PCOS that should be researched further.

Through enrichment analysis, we conclude that PCOS is mainly enriched in the following pathways: oxidative phosphorylation; glycogen biosynthesis; TNF, PPAR, and MAPK signaling; fatty acid metabolic process; lipid catabolic process, etc. One report consistent with our study showed that DEGs in granulosa cells of PCOS are mainly involved in oxidative phosphorylation (Cozzolino et al., 2022). In addition, our observations are similar to the results of a previous study that found that DEGs in granulosa cells are mainly enriched in steroid biosynthesis and metabolism-related signaling, such as glycolysis or gluconeogenesis (Zhao et al., 2021). Moreover, studies from various countries have shown that patients with PCOS tend to have abnormal glucose metabolism, and some of them meet the WHO diagnostic criteria for type 2 diabetes mellitus (T2DM) (Ganie et al., 2016).

PCOS is also considered to be involved in chronic low-grade inflammation, and long-term inflammation may lead to hyperandrogenism unrelated to obesity (Rudnicka et al., 2021; Zhang et al., 2021). Women with PCOS have elevated levels of inflammatory cytokines such as IL-18, IL-1, TNF-α, and IL-6 (Liu et al., 2021; Rudnicka et al., 2021). Moreover, it was recently documented that TNF-α, a member of the adipokine family, may be associated with hyperandrogenism, increased insulin resistance, and obesity, which are common factors in the pathogenesis of PCOS (Escobar-Morreale et al., 2001; Alzamil, 2020; Uzdogan et al., 2021). In addition, in patients with PCOS, several dietary factors can induce oxidative stress and stimulate an inflammatory response, which may be related to insulin resistance in the disease. This oxidative stress may promote hyperandrogenism by reducing levels of the sex hormone-binding globulin (Sun et al., 2021). Besides, peroxisome proliferator-activated receptor-γ (PPAR-γ), a ligand-activated nuclear transcription factor, has been reported to be engaged in the inflammatory response in PCOS (San-Millán and Escobar-Morreale, 2010). One study suggested that the link between obesity and insulin resistance appears to be inflammatory, as pro-inflammatory cytokines are mainly produced by adipose tissue, and alterations in inflammation serum markers appear to be a feature of PCOS itself, unrelated to the PPAR variant (Knebel et al., 2008). Studies by other researchers support these findings, demonstrating that differentially expressed genes are engaged in the MAPK signaling pathway and other signaling pathways related to inflammation (Zhou et al., 2022). MAPK is the key to ovarian follicle development and ovulation. It is well known that activity of MAPK pathway is crucial for meiotic resumption and oocyte expansion in mouse oocytes (Su et al., 2002). Other studies have also found that MAPK is involved in regulating ovulation dysfunction in PCOS patients (Zhou et al., 2020). Another report shows that sterol regulatory element binding protein (SREBP-1), as the promoter of toll-like receptor (TLR)-4 signal transduction, can reprogram fatty acid metabolism, thereby inducing a pro-inflammatory response in PCOS (Wang et al., 2018). Our present results are consistent with these studies.

We constructed a ceRNA network based on the above results. Liu et al. (2019) observed that lncRNA SAMD12-AS1 was highly expressed in liver cancer tissues, and SAMD12-AS1 promoted cell growth and prevented apoptosis. However, if SAMD12-AS1 was knocked down, cell proliferation was inhibited, and apoptosis was enhanced. High lncRNA SAMD12-AS1 expression in glioma cells may increase the invasive and migratory capacity of cells *via* p53, and its high expression in patients may put them at increased risk of developing lymph node or distant metastases (Yu et al., 2019). In addition, lnc-GNAQ-6:1 expression is low in serum exosomes of gastric cancer patients and could be used as a novel diagnostic marker for gastric cancer by increasing sample size (Li et al., 2020b). Although lncRNAs have been studied in different cancerous cells and tissues, similar studies pertaining to PCOS are lacking. Therefore, in-depth studies should be conducted to address the research gap in the future. In addition, it has been shown that miRNAs mediate competition between ceRNAs and thus constitute an additional level of post-transcriptional regulation, which is essential in plenty of biological settings (Figliuzzi et al., 2013). For hsa-miR-508-3p, several studies have discovered that HSPA8 is highly expressed in acute myeloid leukemia, and HSPA8 is associated with hsa-miR-508-3p (Li and Ge, 2021). Another study showed that MiR-508-3p inhibits apoptosis and promotes the proliferation of middle ear cholesteatoma cells *via* the PTEN/PI3K/AKT pathway (Liu and Ma, 2021). For hsa-miR-20b-5p, studies have found that the MALAT1/hsa-mir-20b-5p/TXNIP axis plays an important role in liver inflammation in patients with chronic hepatitis B virus infection and non-alcoholic fatty liver disease (Li J. et al., 2021b). In addition, many studies have investigated the relationship between hsa-miR-20b-5p and cancer (Drobna et al., 2020; Filetti et al., 2021). However, we have not seen hsa-miR-508-3p and hsa-miR-20b-5p reported in studies on PCOS. Therefore, we may be able to further study these two miRNAs *in vivo* and *in vitro* in the future.

Through functional pathway analysis, we found that the ceRNA network potentially regulates multiple signaling pathways, such as the circadian rhythm. There is indeed some evidence to indicate that the development of PCOS is associated with a person’s circadian rhythm. Levels of melatonin in serum (Terzieva et al., 2013; Alizadeh et al., 2021), follicular fluid (Xie et al., 2021), and urine (Shreeve et al., 2013) and its metabolites are elevated in PCOS patients. Physiologically, the female reproductive system is governed by the hypothalamic–pituitary–gonadal axis in a time-dependent manner; thus, an abnormal daily rhythm would contribute to PCOS (Shao et al., 2021). In addition, one study showed night-shift work to be significantly associated with PCOS (Wang et al., 2021).

However, due to the limitation of data sets, we only found one differential circRNA, and the miRNA predicted by circRNA was not one of the DEMs from GSE138572; this needs to be further studied by increasing the datasets. Nevertheless, we identified a gene, HMAG1. HMGA1 has a wide range of roles in insulin resistance, glucose homeostasis, lipid metabolism, and atherosclerosis, and has recently been proposed as a convincing molecule involved in the pathological features of overlapping two diseases, PCOS and cardiovascular disease (De Rosa et al., 2018).

Although bioinformatics analysis is a powerful tool for understanding the pathogenesis of PCOS, further validation of these results at the molecular, cellular, and tissue levels is necessary. In the future, specific mechanisms involved in the regulation of disease development by these lncRNAs, miRNA, and mRNA, and intercellular communication would require further study. Other researchers may develop a more comprehensive ceRNA network based on a larger sample size, better database, and better algorithm. Importantly, more in-depth studies of these RNAs *in vivo* and *in vitro* are required.

Taken together, this study demonstrates the expression profiles of lncRNAs in exosomes, miRNAs, and mRNAs in granulosa cells, and presents ceRNA networks of exosomes and granulosa cells. The aberrant expression of these lncRNAs, miRNAs, and genes may be associated with multiple pathogeneses in patients with PCOS. This novel ceRNA network may prove useful for the study of the pathogenesis and treatment of PCOS.

## Data Availability

The datasets presented in this study can be found in online repositories. The names of the repository/repositories and accession number(s) can be found in the article/Supplementary Material.
