# Supplementary material for: Construction of a ceRNA network in polycystic ovary syndrome (PCOS) driven by exosomal lncRNA
Source: Front Genet. 2022 Nov 4;13:979924. doi: 10.3389/fgene.2022.979924 (PMC9672461; doi:10.3389/fgene.2022.979924)
Supplement: Supplementary file 2 [file DataSheet1.PDF]

Supplementary Material

Supplementary Figures and Tables

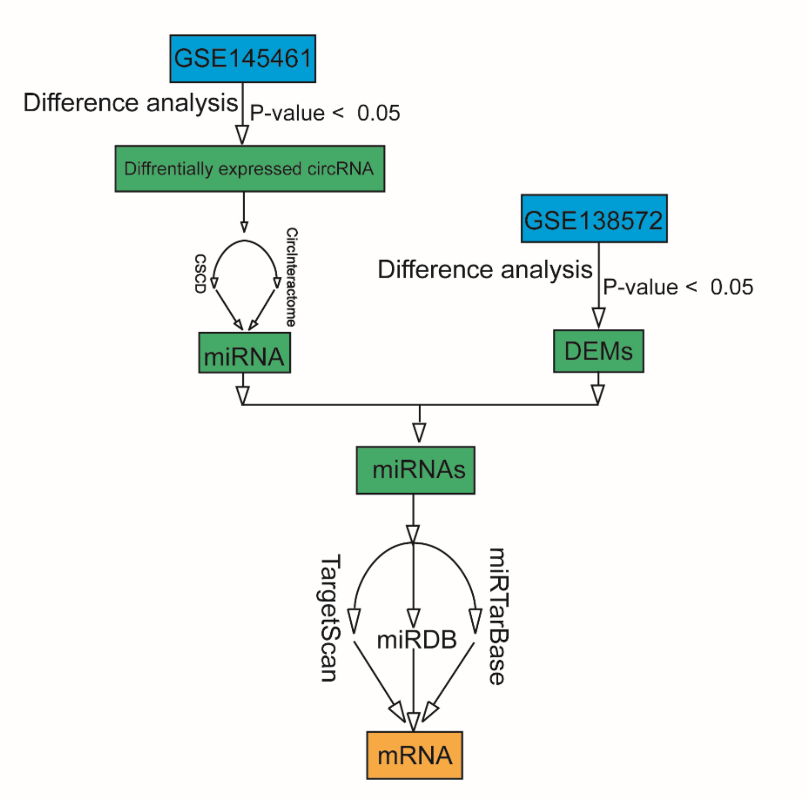

Supplementary Figure 1. The working flow chart of circRNA.

Supplementary Table 1. Screening for differential circRNA using edgeR

id	logFC	logCPM	PValue	FDR	Sig
hsa_circ_0005069	-4.4314	14.5174	0.1067	0.5860	No-sig
hsa_circ_0006877	-4.2661	14.4625	0.1094	0.5860	No-sig
hsa_circ_0033126	-4.1748	14.3877	0.1175	0.5860	No-sig
hsa_circ_0031584	-4.2039	14.4065	0.1253	0.5860	No-sig
hsa_circ_0115004	-4.1769	14.4015	0.1307	0.5860	No-sig
hsa_circ_0002040	-3.9004	14.2966	0.1444	0.5860	No-sig
hsa_circ_0027364	-4.2883	14.4400	0.1481	0.5860	No-sig
hsa_circ_0077438	4.1061	14.3999	0.1509	0.5860	No-sig
hsa_circ_0000024	-4.0638	14.3527	0.1529	0.5860	No-sig
hsa_circ_0124801	4.1011	14.3999	0.1564	0.5860	No-sig
hsa_circ_0001247	-3.9267	14.3167	0.1580	0.5860	No-sig
hsa_circ_0000835	-3.9259	14.3167	0.1606	0.5860	No-sig
hsa_circ_0123217	-4.3338	14.4630	0.1636	0.5860	No-sig
hsa_circ_0007304	-4.1091	14.3633	0.1684	0.5860	No-sig
hsa_circ_0000591	4.0899	14.4198	0.1704	0.5860	No-sig
hsa_circ_0101802	-4.4945	14.5388	0.1713	0.5860	No-sig
hsa_circ_0000072	-3.5006	14.1983	0.1715	0.5860	No-sig
hsa_circ_0001309	-3.5026	14.1983	0.1731	0.5860	No-sig

hsa_circ_0005729	4.0889	14.4198	0.1763	0.5860	No-sig
hsa_circ_0108096	-4.6401	14.6105	0.1775	0.5860	No-sig
hsa_circ_0094296	3.8667	14.3157	0.1797	0.5860	No-sig
hsa_circ_0001788	-4.5115	14.5476	0.1820	0.5860	No-sig
hsa_circ_0098478	-1.9249	14.2770	0.1851	0.5860	No-sig
hsa_circ_0001445	-3.5900	14.2046	0.1861	0.5860	No-sig
hsa_circ_0009144	-4.5348	14.5603	0.1877	0.5860	No-sig
hsa_circ_0003218	4.2011	14.4598	0.1902	0.5860	No-sig
hsa_circ_0000745	-3.6633	14.2320	0.1912	0.5860	No-sig
hsa_circ_0111490	-3.6633	14.2320	0.1936	0.5860	No-sig
hsa_circ_0135062	4.9611	14.8083	0.1983	0.5860	No-sig
hsa_circ_0005585	-3.7925	14.2647	0.1990	0.5860	No-sig
hsa_circ_0000260	-3.8333	14.2706	0.2113	0.5860	No-sig
hsa_circ_0100708	-3.8598	14.2909	0.2125	0.5860	No-sig
hsa_circ_0135237	-3.8597	14.2909	0.2125	0.5860	No-sig
hsa_circ_0140688	5.3697	15.0276	0.2126	0.5860	No-sig
hsa_circ_0084429	-3.8589	14.2909	0.2153	0.5860	No-sig
hsa_circ_0000642	4.2710	14.4788	0.2157	0.5860	No-sig
hsa_circ_0114153	-3.6321	14.2108	0.2167	0.5860	No-sig
hsa_circ_0032943	-3.6316	14.2108	0.2173	0.5860	No-sig
hsa_circ_0004771	4.5147	14.5922	0.2174	0.5860	No-sig

hsa_circ_0058042	-3.6897	14.2529	0.2319	0.5860	No-sig
hsa_circ_0087421	5.4974	15.0728	0.2386	0.5860	No-sig
hsa_circ_0107352	-7.2712	16.3525	0.2390	0.5860	No-sig
hsa_circ_0008571	4.2699	14.5009	0.2420	0.5860	No-sig
hsa_circ_0006258	5.3809	15.5005	0.2490	0.5860	No-sig
hsa_circ_0001633	-3.6037	14.1444	0.2547	0.5860	No-sig
hsa_circ_0000267	-2.8092	14.0985	0.2547	0.5860	No-sig
hsa_circ_0004604	-2.8092	14.0985	0.2547	0.5860	No-sig
hsa_circ_0076179	-2.8092	14.0985	0.2547	0.5860	No-sig
hsa_circ_0138744	-2.8092	14.0985	0.2547	0.5860	No-sig
hsa_circ_0001358	-2.7976	14.0682	0.2547	0.5860	No-sig
hsa_circ_0017701	-2.7976	14.0682	0.2547	0.5860	No-sig
hsa_circ_0125024	-2.7976	14.0682	0.2547	0.5860	No-sig
hsa_circ_0127188	-2.7976	14.0682	0.2547	0.5860	No-sig
hsa_circ_0001206	-3.1551	14.1349	0.2662	0.5860	No-sig
hsa_circ_0002338	3.6397	14.2912	0.2681	0.5860	No-sig
hsa_circ_0032881	4.8984	15.1581	0.2688	0.5860	No-sig
hsa_circ_0006134	-3.5833	14.1908	0.2716	0.5860	No-sig
hsa_circ_0008351	-3.1428	14.1055	0.2733	0.5860	No-sig
hsa_circ_0000826	-5.4066	14.9920	0.2743	0.5860	No-sig

hsa_circ_0079074	-3.1459	14.1055	0.2745	0.5860	No-sig
hsa_circ_0128731	-7.5514	16.2663	0.2855	0.5860	No-sig
hsa_circ_0121400	-3.3006	14.1637	0.2864	0.5860	No-sig
hsa_circ_0133534	5.4039	15.0523	0.2866	0.5860	No-sig
hsa_circ_0085121	-5.9738	15.3532	0.2878	0.5860	No-sig
hsa_circ_0002782	-3.0973	14.1281	0.2896	0.5860	No-sig
hsa_circ_0013143	-5.2194	14.8859	0.2916	0.5860	No-sig
hsa_circ_0002105	3.1048	14.1278	0.2926	0.5860	No-sig
hsa_circ_0102648	3.1026	14.1278	0.2948	0.5860	No-sig
hsa_circ_0006099	5.6982	15.2242	0.2954	0.5860	No-sig
hsa_circ_0008324	3.1013	14.1278	0.2962	0.5860	No-sig
hsa_circ_0006508	-3.7776	14.2585	0.3029	0.5860	No-sig
hsa_circ_0004705	4.9812	14.8132	0.3079	0.5860	No-sig
hsa_circ_0000296	4.9373	14.7965	0.3108	0.5860	No-sig
hsa_circ_0130692	4.9812	14.8132	0.3135	0.5860	No-sig
hsa_circ_0007695	-3.5125	14.1767	0.3144	0.5860	No-sig
hsa_circ_0070476	4.7924	14.7245	0.3210	0.5860	No-sig
hsa_circ_0108513	5.7723	15.2451	0.3247	0.5860	No-sig
hsa_circ_0019170	5.9251	15.3350	0.3287	0.5860	No-sig
hsa_circ_0005087	4.4513	14.5684	0.3304	0.5860	No-sig
hsa_circ_0007755	4.6326	14.6448	0.3310	0.5860	No-sig

hsa_circ_0001492	4.6314	14.6486	0.3321	0.5860	No-sig
hsa_circ_0004575	-3.2755	14.1572	0.3337	0.5860	No-sig
hsa_circ_0114181	5.0491	14.8185	0.3450	0.5915	No-sig
hsa_circ_0001550	-3.2051	14.0815	0.3450	0.5915	No-sig
hsa_circ_0007444	-3.2799	14.1004	0.3515	0.5954	No-sig
hsa_circ_0000607	3.2388	14.9939	0.3616	0.5998	No-sig
hsa_circ_0005567	-3.2188	14.1124	0.3624	0.5998	No-sig
hsa_circ_0007635	-3.3680	14.1483	0.3742	0.6122	No-sig
hsa_circ_0107381	4.5017	15.4458	0.3808	0.6129	No-sig
hsa_circ_0001050	-3.5236	14.1733	0.3835	0.6129	No-sig
hsa_circ_0077495	-3.4975	14.1831	0.3873	0.6129	No-sig
hsa_circ_0005038	-3.7622	14.3595	0.4026	0.6301	No-sig
hsa_circ_0055837	3.4958	15.4946	0.4902	0.7078	No-sig
hsa_circ_0005603	2.6407	14.5051	0.5016	0.7078	No-sig
hsa_circ_0001947	-3.1056	15.5281	0.5020	0.7078	No-sig
hsa_circ_0132814	-3.0835	13.9818	0.5031	0.7078	No-sig
hsa_circ_0001380	-2.5326	14.0609	0.5031	0.7078	No-sig
hsa_circ_0125524	-2.5326	14.0609	0.5031	0.7078	No-sig
hsa_circ_0007577	-2.5209	14.0297	0.5031	0.7078	No-sig
hsa_circ_0093266	-2.5209	14.0297	0.5031	0.7078	No-sig

hsa_circ_0137983	-2.5209	14.0297	0.5031	0.7078	No-sig
hsa_circ_0002579	-2.5545	13.9979	0.5092	0.7078	No-sig
hsa_circ_0016601	-2.5677	13.9979	0.5121	0.7078	No-sig
hsa_circ_0020093	-2.5744	13.9979	0.5140	0.7078	No-sig
hsa_circ_0093522	2.9185	14.0031	0.5286	0.7078	No-sig
hsa_circ_0074052	2.6862	14.0759	0.5293	0.7078	No-sig
hsa_circ_0076792	2.9091	14.0031	0.5301	0.7078	No-sig
hsa_circ_0003520	-2.8814	14.0914	0.5333	0.7078	No-sig
hsa_circ_0036169	-2.8924	14.0914	0.5358	0.7078	No-sig
hsa_circ_0001095	-3.0786	14.0426	0.5451	0.7136	No-sig
hsa_circ_0108628	-3.1449	14.0622	0.5538	0.7185	No-sig
hsa_circ_0000852	3.0358	14.0570	0.5705	0.7271	No-sig
hsa_circ_0103343	3.0356	14.0570	0.5705	0.7271	No-sig
hsa_circ_0006884	-1.7373	14.5131	0.6464	0.8165	No-sig
hsa_circ_0001522	-1.1226	14.5453	0.6597	0.8261	No-sig
hsa_circ_0006705	-1.6970	14.5036	0.6848	0.8501	No-sig
hsa_circ_0007646	-1.8272	14.5231	0.6967	0.8507	No-sig
hsa_circ_0077088	-1.5132	14.2798	0.6971	0.8507	No-sig
hsa_circ_0000119	1.0440	14.4195	0.7704	0.9322	No-sig
hsa_circ_0006139	-1.6255	14.0261	1.0000	1.0000	No-sig
hsa_circ_0008260	-1.6123	14.0261	1.0000	1.0000	No-sig

hsa_circ_0007723	-0.9820	14.1935	1.0000	1.0000	No-sig
hsa_circ_0003051	-1.0088	14.3197	1.0000	1.0000	No-sig
hsa_circ_0006357	2.6447	13.9468	1.0000	1.0000	No-sig
hsa_circ_0000672	-2.4241	13.9175	1.0000	1.0000	No-sig
hsa_circ_0008757	-2.1819	13.9900	1.0000	1.0000	No-sig
hsa_circ_0129676	-2.1703	13.9571	1.0000	1.0000	No-sig
hsa_circ_0107969	-2.1047	13.8953	1.0000	1.0000	No-sig
hsa_circ_0004946	1.8921	13.9585	1.0000	1.0000	No-sig
hsa_circ_0110098	1.8921	13.9585	1.0000	1.0000	No-sig
hsa_circ_0002753	1.8688	13.8878	1.0000	1.0000	No-sig
hsa_circ_0004938	1.8688	13.8878	1.0000	1.0000	No-sig
hsa_circ_0006633	1.8688	13.8878	1.0000	1.0000	No-sig
hsa_circ_0000441	-1.7315	13.9149	1.0000	1.0000	No-sig
hsa_circ_0005129	-1.7315	13.9149	1.0000	1.0000	No-sig
hsa_circ_0006408	-1.7315	13.9149	1.0000	1.0000	No-sig
hsa_circ_0009043	-1.6866	13.8726	1.0000	1.0000	No-sig
hsa_circ_0127796	-1.6866	13.8726	1.0000	1.0000	No-sig
hsa_circ_0078755	-1.1180	13.9063	1.0000	1.0000	No-sig
hsa_circ_0001333	-1.1065	13.8712	1.0000	1.0000	No-sig
hsa_circ_0007976	-1.1065	13.8712	1.0000	1.0000	No-sig

hsa_circ_0130860	-1.1065	13.8712	1.0000	1.0000	No-sig
hsa_circ_0007554	-1.0835	13.8494	1.0000	1.0000	No-sig
hsa_circ_0004826	0.7856	13.9996	1.0000	1.0000	No-sig

Supplementary Table 2. Screening for differential circRNA using limma

id	logFC	AveExpr	t	P.Value	adj.P.Val	B	Sig
hsa_circ_0005069	-1.966852245	-2.107913503	-2.304638688	0.044350712	0.427013964	-4.527899303	Sig
hsa_circ_0006877	-1.877015183	-2.152832034	-2.156502098	0.056923782	0.427013964	-4.535709147	No-sig
hsa_circ_0108096	-2.595586947	-1.793546152	-1.818773461	0.099518019	0.427013964	-4.553952146	No-sig
hsa_circ_0003218	2.060739575	-2.060969838	1.817641968	0.099701166	0.427013964	-4.554013687	No-sig
hsa_circ_0101802	-2.179425743	-2.001626754	-1.812969196	0.100460813	0.427013964	-4.554267833	No-sig
hsa_circ_0123217	-1.581552407	-2.300563422	-1.801637607	0.102325175	0.427013964	-4.554884097	No-sig
hsa_circ_0004771	2.320123697	-1.931277777	1.800938011	0.102441315	0.427013964	-4.554922142	No-sig
hsa_circ_0135062	1.813109962	-2.184784644	1.789666603	0.104329276	0.427013964	-4.555535031	No-sig
hsa_circ_0000591	1.821145991	-2.18076663	1.788361441	0.104549944	0.427013964	-4.555605992	No-sig
hsa_circ_0005729	1.821145991	-2.18076663	1.788361441	0.104549944	0.427013964	-4.555605992	No-sig
hsa_circ_0002105	0.874094154	-2.654292548	1.714380569	0.117779267	0.427013964	-4.559623549	No-sig

hsa_circ_0008324	0.874094154	-2.654292548	1.714380569	0.117779267	0.427013964	-4.559623549	No-sig
hsa_circ_0102648	0.874094154	-2.654292548	1.714380569	0.117779267	0.427013964	-4.559623549	No-sig
hsa_circ_0008571	1.672438807	-2.255120222	1.702033337	0.120130016	0.427013964	-4.560292748	No-sig
hsa_circ_0001633	-1.068636125	-2.557021563	-1.701404933	0.120250785	0.427013964	-4.560326793	No-sig
hsa_circ_0009144	-2.087169143	-2.047755054	-1.700514191	0.12042216	0.427013964	-4.560375049	No-sig
hsa_circ_0000024	-0.767428392	-2.707625429	-1.659542984	0.128547408	0.427013964	-4.562591394	No-sig
hsa_circ_0094296	1.522562595	-2.330058328	1.643391586	0.131883804	0.427013964	-4.563463105	No-sig
hsa_circ_0074052	0.777130918	-2.702774166	1.622717348	0.136267633	0.427013964	-4.564576982	No-sig
hsa_circ_0027364	-1.478449741	-2.352114755	-1.618058712	0.137273264	0.427013964	-4.564827654	No-sig
hsa_circ_0002040	-0.594026595	-2.794326328	-1.609764781	0.13908002	0.427013964	-4.56527362	No-sig
hsa_circ_0005585	-0.565428372	-2.808625439	-1.590382825	0.143384991	0.427013964	-4.56631414	No-sig
hsa_circ_0077438	1.74009399	-2.22129263	1.580252292	0.145681856	0.427013964	-4.566857021	No-sig
hsa_circ_0124801	1.74009399	-2.22129263	1.580252292	0.145681856	0.427013964	-4.566857021	No-sig
hsa_circ_0140688	1.781946718	-2.200366266	1.569211841	0.148222078	0.427013964	-4.56744785	No-sig
hsa_circ_0002338	1.412271134	-2.385204058	1.551010063	0.1524955	0.427013964	-4.56841996	No-sig

hsa_circ_0000642	1.897810428	-2.142434411	1.540584991	0.154991676	0.427013964	-4.568975577	No-sig
hsa_circ_0031584	-1.153381439	-2.514648906	-1.537752871	0.155675971	0.427013964	-4.569126366	No-sig
hsa_circ_0084429	-0.667130918	-2.757774166	-1.520457127	0.159912772	0.427013964	-4.570045767	No-sig
hsa_circ_0100708	-0.667130918	-2.757774166	-1.520457127	0.159912772	0.427013964	-4.570045767	No-sig
hsa_circ_0135237	-0.667130918	-2.757774166	-1.520457127	0.159912772	0.427013964	-4.570045767	No-sig
hsa_circ_0001788	-1.861733603	-2.160472824	-1.514414622	0.161416571	0.427013964	-4.570366359	No-sig
hsa_circ_0000745	-0.493729122	-2.844475064	-1.50401951	0.164032524	0.427013964	-4.570917108	No-sig
hsa_circ_0111490	-0.493729122	-2.844475064	-1.50401951	0.164032524	0.427013964	-4.570917108	No-sig
hsa_circ_0000260	-0.685069932	-2.748804659	-1.479291638	0.170404235	0.427013964	-4.572223096	No-sig
hsa_circ_0000072	-0.47559131	-2.85354397	-1.472090979	0.172299579	0.427013964	-4.572602248	No-sig
hsa_circ_0001309	-0.47559131	-2.85354397	-1.472090979	0.172299579	0.427013964	-4.572602248	No-sig
hsa_circ_0001445	-0.47559131	-2.85354397	-1.472090979	0.172299579	0.427013964	-4.572602248	No-sig
hsa_circ_0087421	1.315307898	-2.433685676	1.452739203	0.177483933	0.427013964	-4.573618507	No-sig
hsa_circ_0033126	-1.305047944	-2.438815653	-1.452369389	0.177584303	0.427013964	-4.573637889	No-sig

hsa_circ_0007695	-0.475888783	-2.853395234	-1.426414361	0.184751745	0.427013964	-4.57499426	No-sig
hsa_circ_0032943	-0.656769182	-2.762955034	-1.424173865	0.185381927	0.427013964	-4.575110976	No-sig
hsa_circ_0114153	-0.656769182	-2.762955034	-1.424173865	0.185381927	0.427013964	-4.575110976	No-sig
hsa_circ_0121400	-0.44729056	-2.867694345	-1.413326705	0.188458965	0.427013964	-4.575675184	No-sig
hsa_circ_0002782	-0.43559131	-2.87354397	-1.385810905	0.196460434	0.427013964	-4.577099751	No-sig
hsa_circ_0000835	-1.034946153	-2.573866549	-1.385794114	0.196465403	0.427013964	-4.577100617	No-sig
hsa_circ_0001247	-1.034946153	-2.573866549	-1.385794114	0.196465403	0.427013964	-4.577100617	No-sig
hsa_circ_0001206	-0.385754248	-2.898462501	-1.3719068	0.200612133	0.427013964	-4.577815801	No-sig
hsa_circ_0115004	-1.368106921	-2.407286165	-1.34518865	0.208798097	0.427013964	-4.579184161	No-sig
hsa_circ_0008351	-0.37559131	-2.90354397	-1.344209984	0.209103193	0.427013964	-4.579234087	No-sig
hsa_circ_0079074	-0.37559131	-2.90354397	-1.344209984	0.209103193	0.427013964	-4.579234087	No-sig
hsa_circ_0058042	-0.994946153	-2.593866549	-1.328032299	0.214200902	0.427013964	-4.580057287	No-sig
hsa_circ_0000267	-0.357453498	-2.912612876	-1.315698362	0.218156776	0.427013964	-4.580682195	No-sig
hsa_circ_0001358	-0.357453498	-2.912612876	-1.315698362	0.218156776	0.427013964	-4.580682195	No-sig
hsa_circ_0004604	-0.357453498	-2.912612876	-1.315698362	0.218156776	0.427013964	-4.580682195	No-sig

hsa_circ_0017701	-0.357453498	-2.912612876	-1.315698362	0.218156776	0.427013964	-4.580682195	No-sig
hsa_circ_0076179	-0.357453498	-2.912612876	-1.315698362	0.218156776	0.427013964	-4.580682195	No-sig
hsa_circ_0125024	-0.357453498	-2.912612876	-1.315698362	0.218156776	0.427013964	-4.580682195	No-sig
hsa_circ_0127188	-0.357453498	-2.912612876	-1.315698362	0.218156776	0.427013964	-4.580682195	No-sig
hsa_circ_0138744	-0.357453498	-2.912612876	-1.315698362	0.218156776	0.427013964	-4.580682195	No-sig
hsa_circ_0007304	-1.440778306	-2.370950472	-1.312116575	0.219316895	0.427013964	-4.580863219	No-sig
hsa_circ_0001380	-0.345754248	-2.918462501	-1.288349604	0.227145247	0.427013964	-4.582059127	No-sig
hsa_circ_0125524	-0.345754248	-2.918462501	-1.288349604	0.227145247	0.427013964	-4.582059127	No-sig
hsa_circ_0007577	-0.329152748	-2.926763251	-1.260069264	0.236759138	0.427013964	-4.583469681	No-sig
hsa_circ_0093266	-0.329152748	-2.926763251	-1.260069264	0.236759138	0.427013964	-4.583469681	No-sig
hsa_circ_0137983	-0.329152748	-2.926763251	-1.260069264	0.236759138	0.427013964	-4.583469681	No-sig
hsa_circ_0000119	0.953816263	-2.285278746	1.250636727	0.240038934	0.427013964	-4.583937007	No-sig
hsa_circ_0008757	-0.317453498	-2.932612876	-1.232789739	0.246345875	0.427013964	-4.584816747	No-sig
hsa_circ_0004575	-0.522554544	-2.830062353	-1.196889307	0.259439388	0.427013964	-4.586567915	No-sig

hsa_circ_0003520	-0.349152748	-2.916763251	-1.128953188	0.285736359	0.427013964	-4.589808723	No-sig
hsa_circ_0036169	-0.349152748	-2.916763251	-1.128953188	0.285736359	0.427013964	-4.589808723	No-sig
hsa_circ_0006508	-1.40561762	-2.388530815	-1.105972981	0.295090853	0.427013964	-4.590881653	No-sig
hsa_circ_0107352	-1.40561762	-2.388530815	-1.105972981	0.295090853	0.427013964	-4.590881653	No-sig
hsa_circ_0108513	1.40561762	-2.388530815	1.105972981	0.295090853	0.427013964	-4.590881653	No-sig
hsa_circ_0133534	1.40561762	-2.388530815	1.105972981	0.295090853	0.427013964	-4.590881653	No-sig
hsa_circ_0006134	-1.28975391	-2.44646267	-1.104315238	0.295774763	0.427013964	-4.590958572	No-sig
hsa_circ_0085121	-1.28975391	-2.44646267	-1.104315238	0.295774763	0.427013964	-4.590958572	No-sig
hsa_circ_0000296	1.206048454	-2.488315398	1.102738049	0.296426579	0.427013964	-4.591031693	No-sig
hsa_circ_0000826	-1.206048454	-2.488315398	-1.102738049	0.296426579	0.427013964	-4.591031693	No-sig
hsa_circ_0001050	-1.206048454	-2.488315398	-1.102738049	0.296426579	0.427013964	-4.591031693	No-sig
hsa_circ_0019170	1.206048454	-2.488315398	1.102738049	0.296426579	0.427013964	-4.591031693	No-sig
hsa_circ_0070476	1.108135516	-2.537271867	1.100328353	0.297424601	0.427013964	-4.591143294	No-sig
hsa_circ_0007444	-1.108135516	-2.537271867	-1.100328353	0.297424601	0.427013964	-4.591143294	No-sig
hsa_circ_0013143	-1.108135516	-2.537271867	-1.100328353	0.297424601	0.427013964	-4.591143294	No-sig

hsa_circ_0001550	-1.030369787	-2.576154732	-1.097821593	0.298465581	0.427013964	-4.591259243	No-sig
hsa_circ_0001492	1.022333758	-2.580172746	1.097525055	0.298588911	0.427013964	-4.59127295	No-sig
hsa_circ_0108628	-1.014297729	-2.584190761	-1.097220597	0.298715575	0.427013964	-4.59128702	No-sig
hsa_circ_0005087	0.95154765	-2.6155658	1.094539635	0.299832734	0.427013964	-4.59141082	No-sig
hsa_circ_0114181	0.95154765	-2.6155658	1.094539635	0.299832734	0.427013964	-4.59141082	No-sig
hsa_circ_0001095	-0.95154765	-2.6155658	-1.094539635	0.299832734	0.427013964	-4.59141082	No-sig
hsa_circ_0132814	-0.842069029	-2.670305111	-1.088186236	0.302493079	0.427013964	-4.591703508	No-sig
hsa_circ_0006139	-0.842069029	-2.670305111	-1.088186236	0.302493079	0.427013964	-4.591703508	No-sig
hsa_circ_0008260	-0.842069029	-2.670305111	-1.088186236	0.302493079	0.427013964	-4.591703508	No-sig
hsa_circ_0077495	-0.739483377	-2.721597937	-1.079293289	0.30624729	0.427013964	-4.592111527	No-sig
hsa_circ_0004946	0.733633747	-2.724522752	1.078666499	0.306513237	0.427013964	-4.592140211	No-sig
hsa_circ_0110098	0.733633747	-2.724522752	1.078666499	0.306513237	0.427013964	-4.592140211	No-sig
hsa_circ_0000672	-0.727784117	-2.727447567	-1.07802418	0.306785957	0.427013964	-4.592169596	No-sig
hsa_circ_0107969	-0.709646316	-2.736516467	-1.075928552	0.307677027	0.427013964	-4.592265395	No-sig

hsa_circ_0128731	-1.339179059	-2.421750096	-1.042641928	0.322097334	0.427013964	-4.593772127	No-sig
hsa_circ_0006099	1.223315348	-2.479681951	1.035614003	0.325206372	0.427013964	-4.594086581	No-sig
hsa_circ_0001522	-0.854065755	-2.17211394	-1.034909647	0.32551921	0.427013964	-4.594118024	No-sig
hsa_circ_0009043	-0.492192808	-2.845243221	-1.030677763	0.327403567	0.427013964	-4.594306664	No-sig
hsa_circ_0127796	-0.492192808	-2.845243221	-1.030677763	0.327403567	0.427013964	-4.594306664	No-sig
hsa_circ_0000852	0.492192808	-2.845243221	1.030677763	0.327403567	0.427013964	-4.594306664	No-sig
hsa_circ_0007635	-0.492192808	-2.845243221	-1.030677763	0.327403567	0.427013964	-4.594306664	No-sig
hsa_circ_0103343	0.492192808	-2.845243221	1.030677763	0.327403567	0.427013964	-4.594306664	No-sig
hsa_circ_0005038	-1.139609892	-2.521534679	-1.029569017	0.327898619	0.427013964	-4.594356008	No-sig
hsa_circ_0004705	1.090653423	-2.546012914	1.025567434	0.329689992	0.427013964	-4.594533823	No-sig
hsa_circ_0130692	1.090653423	-2.546012914	1.025567434	0.329689992	0.427013964	-4.594533823	No-sig
hsa_circ_0007554	-0.463892058	-2.859393596	-1.020160871	0.332121972	0.427013964	-4.594773385	No-sig
hsa_circ_0076792	0.463892058	-2.859393596	1.020160871	0.332121972	0.427013964	-4.594773385	No-sig
hsa_circ_0093522	0.463892058	-2.859393596	1.020160871	0.332121972	0.427013964	-4.594773385	No-sig
hsa_circ_0007755	0.963931226	-2.609374012	1.013163746	0.335289315	0.427271339	-4.595082247	No-sig

hsa_circ_0004826	0.667195186	-2.691303471	0.979080879	0.35103936	0.440246765	-4.596567296	No-sig
hsa_circ_0006357	0.381901346	-2.900388952	0.977918489	0.351585958	0.440246765	-4.596617364	No-sig
hsa_circ_0107381	1.183109391	-2.335208556	0.96454919	0.357917585	0.444311484	-4.597190418	No-sig
hsa_circ_0005567	-0.311312409	-2.935683421	-0.919757526	0.379734002	0.467364926	-4.599071699	No-sig
hsa_circ_0002753	0.28493811	-2.94887057	0.890133753	0.394674084	0.473608901	-4.600281975	No-sig
hsa_circ_0004938	0.28493811	-2.94887057	0.890133753	0.394674084	0.473608901	-4.600281975	No-sig
hsa_circ_0006633	0.28493811	-2.94887057	0.890133753	0.394674084	0.473608901	-4.600281975	No-sig
hsa_circ_0000607	0.783282794	-2.468683293	0.840002151	0.42088366	0.500886339	-4.602265359	No-sig
hsa_circ_0005603	0.783282794	-2.535121854	0.82748538	0.427608854	0.504718648	-4.602747413	No-sig
hsa_circ_0007646	-0.812855136	-2.192719249	-0.816366303	0.433643577	0.507680285	-4.603171109	No-sig
hsa_circ_0002579	-0.192877124	-2.994901063	-0.733573677	0.480351759	0.548973438	-4.606186849	No-sig
hsa_circ_0016601	-0.192877124	-2.994901063	-0.733573677	0.480351759	0.548973438	-4.606186849	No-sig
hsa_circ_0020093	-0.192877124	-2.994901063	-0.733573677	0.480351759	0.548973438	-4.606186849	No-sig
hsa_circ_0078755	-0.164576374	-3.009051438	-0.662788989	0.522714256	0.588053538	-4.608559961	No-sig

hsa_circ_0129676	-0.164576374	-3.009051438	-0.662788989	0.522714256	0.588053538	-4.608559961	No-sig
hsa_circ_0006258	0.875287001	-2.189804067	0.650158974	0.530501722	0.592187969	-4.60896248	No-sig
hsa_circ_0000441	-0.152877124	-3.014901063	-0.62965835	0.543286231	0.592675889	-4.609601956	No-sig
hsa_circ_0005129	-0.152877124	-3.014901063	-0.62965835	0.543286231	0.592675889	-4.609601956	No-sig
hsa_circ_0006408	-0.152877124	-3.014901063	-0.62965835	0.543286231	0.592675889	-4.609601956	No-sig
hsa_circ_0006884	-0.589876222	-2.068617397	-0.619119403	0.549927148	0.595409845	-4.609923934	No-sig
hsa_circ_0032881	0.644243458	-2.305325838	0.5938381	0.566044857	0.60828701	-4.610677311	No-sig
hsa_circ_0007723	0.47291628	-2.497427987	0.555301299	0.591110373	0.630517732	-4.61177314	No-sig
hsa_circ_0098478	-0.208791012	-2.702006009	-0.512175901	0.61984637	0.656307922	-4.612922269	No-sig
hsa_circ_0077088	0.257744964	-2.48657836	0.354095327	0.730750938	0.762703668	-4.61640195	No-sig
hsa_circ_0055837	0.505616736	-1.996462228	0.353856568	0.730924349	0.762703668	-4.616406303	No-sig
hsa_circ_0001333	-0.066438562	-3.058120344	-0.310288267	0.762825557	0.779055888	-4.617153592	No-sig
hsa_circ_0007976	-0.066438562	-3.058120344	-0.310288267	0.762825557	0.779055888	-4.617153592	No-sig
hsa_circ_0130860	-0.066438562	-3.058120344	-0.310288267	0.762825557	0.779055888	-4.617153592	No-sig
hsa_circ_0003051	0.235591309	-2.481351163	0.29454196	0.774474844	0.785382941	-4.617400486	No-sig

hsa_circ_0001947	-0.357521935	-1.88220887	-0.242824656	0.813136801	0.818823073	-4.618123592	No-sig
hsa_circ_0006705	-0.039644274	-2.343733371	-0.050123706	0.961028451	0.961028451	-4.619604712	No-sig

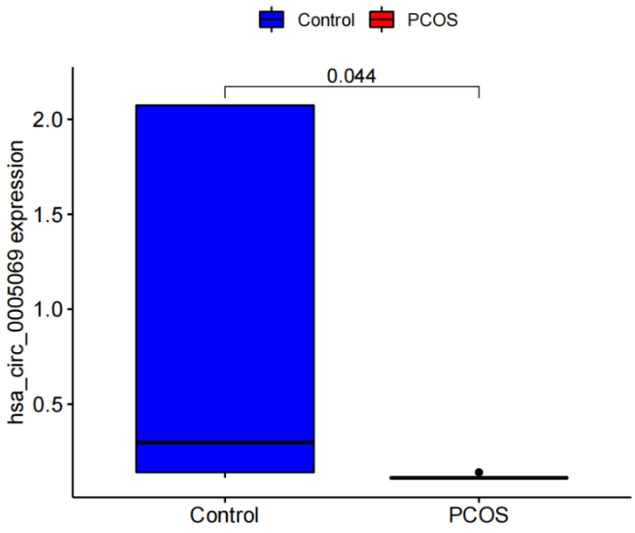

Supplementary Figure 2. Box plot of differential circRNA.

Supplementary Table 3. Prediction of targeting miRNAs of circRNA

circinteractome	CSCD
hsa-miR-1184	hsa-miR-103-5p
hsa-miR-1205	hsa-miR-1184
hsa-miR-1243	hsa-miR-1202
hsa-miR-1288	hsa-miR-3972
hsa-miR-1304	hsa-miR-1205
hsa-miR-145	hsa-miR-1226-5p
hsa-miR-1825	hsa-miR-1245-3p
hsa-miR-186	hsa-miR-1256
hsa-miR-370	hsa-miR-1273-5p
hsa-miR-375	hsa-miR-1273h-3p
hsa-miR-433	hsa-miR-1293
hsa-miR-526b	hsa-miR-4483
hsa-miR-553	hsa-miR-130-3p
hsa-miR-578	hsa-miR-301-3p
hsa-miR-580	hsa-miR-3666
hsa-miR-609	hsa-miR-4295
hsa-miR-640	hsa-miR-454-3p
hsa-miR-649	hsa-miR-140-5p

hsa-miR-885-3p	hsa-miR-17-3p
	hsa-miR-182-5p
	hsa-miR-202-5p
	hsa-miR-203-5p
	hsa-miR-219-5p
	hsa-miR-4782-3p
	hsa-miR-6766-3p
	hsa-miR-29b-2-5p
	hsa-miR-3146
	hsa-miR-3154
	hsa-miR-3158-3p
	hsa-miR-3158-5p
	hsa-miR-3179
	hsa-miR-3182
	hsa-miR-323-5p
	hsa-miR-3616-3p
	hsa-miR-363-5p
	hsa-miR-6745
	hsa-miR-3653-3p
	hsa-miR-3678-3p
	hsa-miR-3685

hsa-miR-384

hsa-miR-4445-5p

hsa-miR-4446-3p

hsa-miR-4639-5p

hsa-miR-4642

hsa-miR-4649-3p

hsa-miR-4656

hsa-miR-4663

hsa-miR-4675

hsa-miR-4741

hsa-miR-4721

hsa-miR-4769-3p

hsa-miR-6817-5p

hsa-miR-494-3p

hsa-miR-5006-5p

hsa-miR-500a-3p

hsa-miR-508-3p

hsa-miR-5088-5p

hsa-miR-544a

hsa-miR-5571-3p

hsa-miR-6508-5p

hsa-miR-8067

hsa-miR-6734-5p

hsa-miR-6738-3p

hsa-miR-6756-5p

hsa-miR-6766-5p

hsa-miR-6815-3p

hsa-miR-6829-5p

hsa-miR-6834-5p

hsa-miR-6882-3p

hsa-miR-7160-5p

hsa-miR-7162-3p

hsa-miR-7515

hsa-miR-762

hsa-miR-4492

hsa-miR-4498

hsa-miR-5001-5p

hsa-miR-7975

hsa-miR-8077

hsa-miR-876-3p

hsa-miR-96-5p

hsa-miR-1271-5p

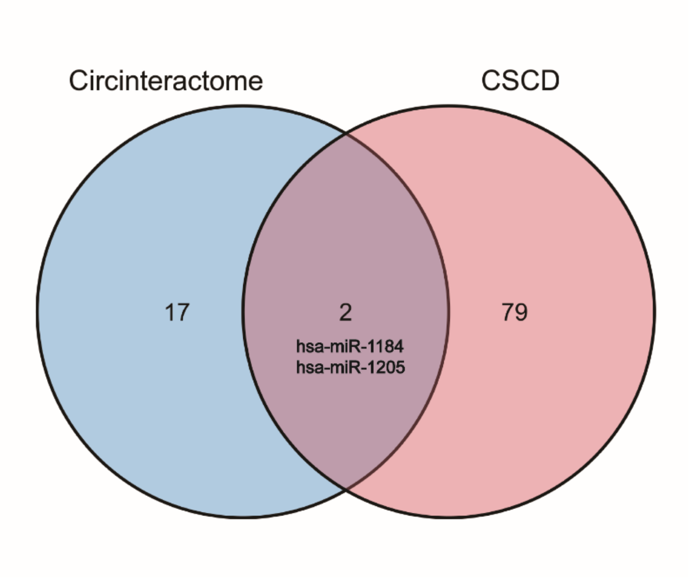

Supplementary Figure 3. The predicted miRNAs from CSCD and circinteractome databases were taken as intersection.

Supplementary Table 4. Intersection of targeted miRNAs

hsa-miR-1184
hsa-miR-1205

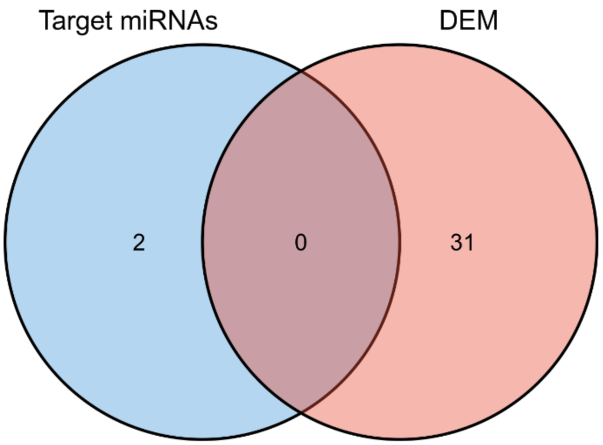

Supplementary Figure 4. Targeting miRNA predicted by CircInteractome and CSCD intersects with DEM.

Supplementary Table5. Prediction of miRNA targeting mRNA

miRNA	Gene	miRDB	miRTarBase	TargetScan	Sum
hsa-miR-1205	TNFAIP1	1	1	1	3
hsa-miR-1184	HIST1H2BK	1	1	1	3
hsa-miR-1205	POLDIP2	1	1	1	3
hsa-miR-1205	NUP35	1	1	1	3
hsa-miR-1205	SMARCE1	1	1	1	3
hsa-miR-1184	DCC	1	1	1	3
hsa-miR-1205	PPP2R5D	1	1	1	3
hsa-miR-1205	COPS7B	1	1	1	3
hsa-miR-1184	HMGA1	1	1	1	3
hsa-miR-1184	TMEM109	1	1	1	3
hsa-miR-1205	FGFR1OP	1	1	1	3
hsa-miR-1205	TRMT6	1	1	1	3
hsa-miR-1205	SKI	1	1	1	3
hsa-miR-1205	CRKL	1	1	1	3
hsa-miR-1184	FAM98A	1	1	1	3
hsa-miR-1184	ENPP2	1	1	1	3
hsa-miR-1184	ZDHHC6	1	1	1	3
hsa-miR-1205	PTBP1	1	1	1	3

hsa-miR-1205	SBNO1	1	1	1	3
hsa-miR-1184	NRBP1	1	1	1	3
hsa-miR-1205	RAB1A	1	1	1	3
hsa-miR-1184	PEA15	1	1	1	3
hsa-miR-1205	KCTD2	1	1	1	3
hsa-miR-1205	CAPN1	1	1	1	3
hsa-miR-1184	GPCPD1	1	1	1	3
hsa-miR-1184	LSM14A	1	1	1	3
hsa-miR-1205	MTA1	1	1	1	3
hsa-miR-1205	RNF11	1	1	1	3
hsa-miR-1205	FEM1A	1	1	1	3
hsa-miR-1184	DRAXIN	1	1	1	3
hsa-miR-1205	UBE2H	1	1	1	3
hsa-miR-1184	IBA57	1	1	1	3
hsa-miR-1184	MIS18BP1	1	1	1	3
hsa-miR-1205	ENPP2	1	1	1	3
hsa-miR-1205	FAM98A	1	1	1	3
hsa-miR-1184	NUMB	1	1	1	3
hsa-miR-1205	PICALM	1	1	1	3
hsa-miR-1205	ZDHHC6	1	1	1	3
hsa-miR-1184	ATG12	1	1	1	3

hsa-miR-1184	SBNO1	1	1	1	3
hsa-miR-1205	F2RL3	1	1	1	3
hsa-miR-1184	TMEM170A	1	1	1	3
hsa-miR-1184	SMARCE1	1	1	1	3
hsa-miR-1205	JPH2	1	1	1	3
hsa-miR-1205	NR6A1	1	1	1	3
hsa-miR-1184	VGLL4	1	1	1	3
hsa-miR-1184	POLDIP2	1	1	1	3
hsa-miR-1205	HIST1H2BK	1	1	1	3
hsa-miR-1205	SLC35F6	1	1	1	3
hsa-miR-1205	MSI1	1	1	1	3
hsa-miR-1184	SRSF9	1	1	1	3
hsa-miR-1184	LRPAP1	1	1	1	3
hsa-miR-1184	DICER1	1	1	1	3
hsa-miR-1205	TOR2A	1	1	1	3
hsa-miR-1184	GCC1	1	1	1	3
hsa-miR-1205	LSM14A	1	1	1	3
hsa-miR-1184	RNF11	1	1	1	3
hsa-miR-1205	ANKS1A	1	1	1	3
hsa-miR-1205	GPATCH3	1	1	1	3

hsa-miR-1184	GGA2	1	1	1	3
hsa-miR-1184	RAB1A	1	1	1	3
hsa-miR-1184	IDH3A	1	1	1	3
hsa-miR-1184	ATOH8	1	1	1	3

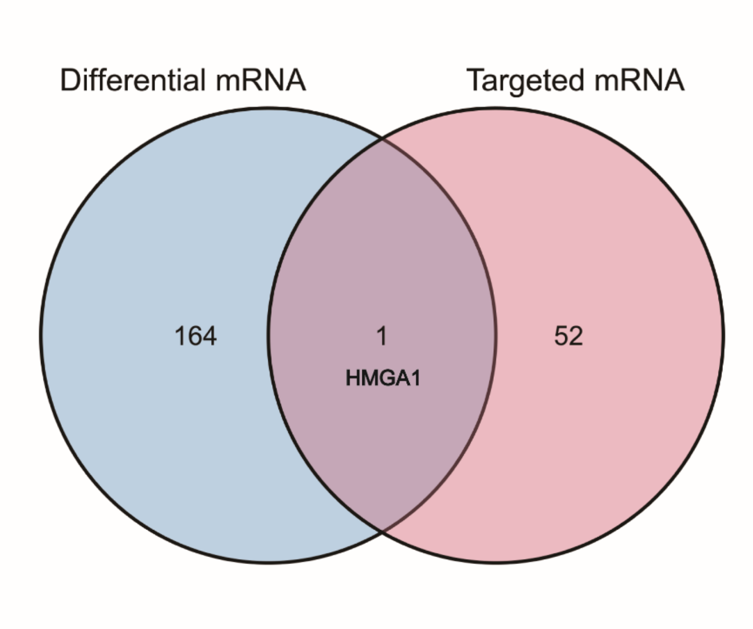

Supplementary Figure 5. The intersecting mRNAs of GSE193123 and GSE155489 are taken to intersect with the target mRNA of the miRNA corresponding to the circRNA in GSE145461.

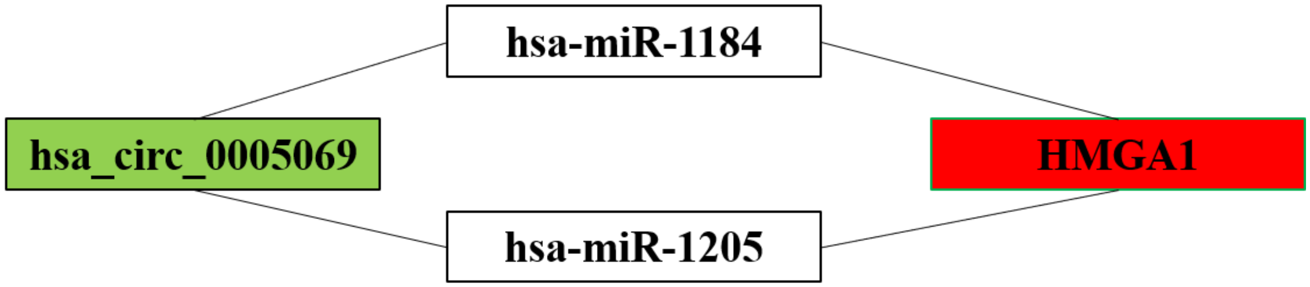

Supplementary Figure 6. CircRNA-related network.
